# Substrate Rigidity Effect on CAD/CAM Restorations at Different Thicknesses

**DOI:** 10.1055/s-0042-1757910

**Published:** 2022-12-13

**Authors:** César Rogério Pucci, Ana Paula Valente Pinho Mafetano, Alexandre Luiz Souto Borges, Guilherme Schmitt de Andrade, Amanda Maria de Oliveira Dal Piva, Cornelis J. Kleverlaan, João Paulo Mendes Tribst

**Affiliations:** 1 Department of Restorative Dentistry, Institute of Science and Technology, São Paulo State University (Unesp), São José dos Campos, Brazil; 2 Department of Dentistry, Universidade Estadual do Oeste do Paraná (UNIOESTE), Cascavel, Brazil; 3 Department of Dental Materials Science, Academic Centre for Dentistry Amsterdam (ACTA), Universiteit van Amsterdam and Vrije Universiteit, Amsterdam, The Netherlands; 4 Department of Oral Regenerative Medicine, Academic Centre for Dentistry Amsterdam (ACTA), Universiteit van Amsterdam and Vrije Universiteit, Amsterdam, The Netherlands

**Keywords:** adhesion, cyclic loading, dental ceramics, finite element analysis, monolithic restorations.

## Abstract

**Objectives** This article evaluated the effect of substrates rigidities on the post-fatigue fracture resistance of adhesively cemented simplified restorations in lithium disilicate glass ceramic.

**Methods** Precrystalized computer-aided design/computer-aided manufacturing ceramic blocks were processed into disc-shaped specimens ( *n* = 10, *Ø* = 10 mm), mimicking a simplified restoration at two thicknesses (0.5 and 1.0 mm). Thereafter, the discs were cemented onto different base substrates (dentin analogue [control], dentin analogue with a central core build-up of resin composite [RC], or glass ionomer cement [GIC]). The specimens were subjected to mechanical cycling in a chewing simulator (100 N, 1 × 10 ^6^ cycles, 4 Hz) and then subjected to thermocycling aging (10,000 cycles, 5/37/55°C, 30 seconds). After the fatigue protocol, the specimens were loaded until failure (N) in a universal testing machine. Finite element analysis calculated the first principal stress at the center of the adhesive interface.

**Results** The results showed that “restoration thickness,” “type of substrate,” and their interaction were statistically significant (one-way analysis of variance; *p* < 0.001). Regardless the restoration thickness a higher fracture load was observed for specimens cemented to dentin analogue. Among the base materials, RC build-up presented the highest fracture load and lower stress magnitude for both restoration thicknesses in comparison with GIC build-up. The 0.5-mm restoration showed higher stress peak and lower fracture load when submitted to the compressive test.

**Conclusion** More flexible base material reduces the fracture load and increases the stress magnitude of adhesively cemented lithium disilicate restorations regardless the ceramic thickness. Therefore, more rigid substrates are suggested to be used to prevent restoration mechanical failures.

## Introduction

The contemporary computer-aided design/computer-aided manufacturing (CAD/CAM) dental ceramic materials improved the alternatives and reliability for indirect restoration of the missing dentin and enamel tissues. CAD/CAM ceramics are available in crystalized or precrystalized blocks with different compositions 1 promoting optimal mechanical properties. 2 3 4 5 6 7 Biomaterials, such as reinforced glass ceramics, are one of the most reliable options to be applied as monolithic restorations with acceptable resistance and optical properties. 8 9 In addition, the high presence of silica and glassy matrix in the volume of these materials is associated with improved translucency and optimal esthetics for prosthetic treatment. 9 Additionally, it is well known that indirect restorations adhesively cemented to enamel presents reliable bond strength and long-term durability. 1 2 3 4 5 6 7 8 9

Despite the success of adhesive dentistry, it is not rare that after the tooth preparation, the substrate is not only in enamel, thus it is not always in the most suitable condition for the best bonding procedure. 10 In cases, for example, after endodontic treatments, it is common for the clinician to fill the access cavity with direct restorative materials (e.g., glass ionomer cement [GIC], resin composite [RC]). Thus, during the prosthetic treatment, the ceramic restorations will be adhesively cemented on different foundation substrates (enamel and dentin, only dentin, dentin partially restored with filling material) with a large range of mechanical properties. 8 9 The base material (below the ceramic) can modify the mechanical response of the restorations. 10 11 Therefore, the restorative material for core build-up should not only be chosen to promote retention to the restorations but also to promote adequate support to the dental ceramic. Previous reports showed that rigid base materials result in less deformations and subsequently higher restoration resistance. 12 13 However, with the development of the adhesive dentistry the most used base materials are RC or GIC. Both materials have a low elastic modulus; however, there is a different bond strength to the resin cement and substrate. 10

According to the literature, the most suitable material to replace the dentin tissue is still controversial. 14 Moreover to the substrate mechanical property, the restoration thickness plays an important role in the fatigue survival and long-term clinical success. 9 According to the principles of the minimal tooth preparation, the maximum amount of sound dental tissue should be kept and unnecessary preparation should be avoided. Based on this principle, several studies have been developed aiming to elucidate how dental materials applied with minimal thickness stand out against the chewing loads. 9 10 11 12 13 15 16

Brittle materials such as dental ceramics are prone to fail due to the slow crack propagation in areas with high tensile stress magnitude. 4 17 Because of that, the knowledge about the stress concentration during the functional loading are useful to demonstrate the regions susceptible to failure that can be coincident with failure origin demonstrated by fractographic features. 9 One of the most reliable methods to investigate the stress field is the finite element analysis (FEA). 2 3 This methodology is a biomechanical tool based on a numerical calculation considering the setup of geometrical model and the mechanical properties of the investigated materials. FEA can show, with qualitative and quantitative results, the biomechanical behavior of different conditions in direct and indirect restorations. 2 9 11 14 17 18

The aim of this study was to evaluate the effect of different substrate stiffness (dentin analogue, dentin analogue, and a core in GIC or composite resin [CR]) on the fracture load after fatigue and on the stress magnitude of conventional (1.0 mm) and conservative (0.5 mm) adhesively cemented lithium disilicate restorations. The hypotheses consisted that (1) the different substrates would affect the fracture load and stress magnitude in the restoration, and that (2) the restoration thicknesses would affect fracture load and stress concentration during load application.

## Materials and Methods

Specimens were prepared and divided into six groups; dentin analogue (D), dentin analogue and a core in GIC or CR combined with 1.0 mm (conventional restoration) or 0.5 mm (conservative restoration) lithium disilicate glass-ceramic disc. The specimens were subjected to mechanical and thermal fatigue and subsequently loaded till failure. The experimental details are described below. The group distribution is described in Table 1 .

**Table 1:** Groups' distribution according to the ceramic thickness and foundation substrate

Group	Ceramic thickness	Foundation substrate	Fracture load ± SD	Tukey test (95%) a
D0.5	0.5	Dentin analogue	1393 ± 301	B
D1.0	1.0		1789 ± 233	A
GIC0.5	0.5	Glass ionomer cement	428 ± 93	D
GIC1.0	1.0		541 ± 173	D
RC0.5	0.5	Resin composite	1043 ± 339	C
RC1.0	1.0		1187 ± 317	BC

Note: Average fracture load (N) ± standard deviation (SD) according to the material and ceramic thickness.

a Means that do not share a letter are significantly different.

### Ceramic Restorations

Lithium disilicate glass-ceramic blocks (IPS e.max CAD, Ivoclar Vivadent, Schaan, Liechtenstein) were rounded in an automatic orbital sander (Ecomet Polisher, Buehler LTD, United States) using wet sandpaper with grain size #600. The resultant cylindrical rollers were cut into discs under constant water irrigation ( *n* = 40) using a precision cutting machine (Isomet 1000, Buehler LTD). All discs were finished in the automatic polishing machine (Ecomet Polisher, Buehler LTD) with an applied force of 30 N at a speed of 450 revolutions per minute for 5 minutes with progressive grits of silicon carbide grinding paper (#120, #240, #320, and #600) under constant water cooling. The 10-mm diameter discs were divided into two different thickness groups with final thickness dimensions of 1.0 mm (conventional restoration) or 0.5 mm (conservative restoration). All specimens were cleaned with isopropyl alcohol in an ultrasonic bath (5 minutes) and then crystallized in a specific oven (Programat P700, Ivoclar Vivadent) according to the manufacturer's instructions.

### Base Substrates

**Fig. 1:**
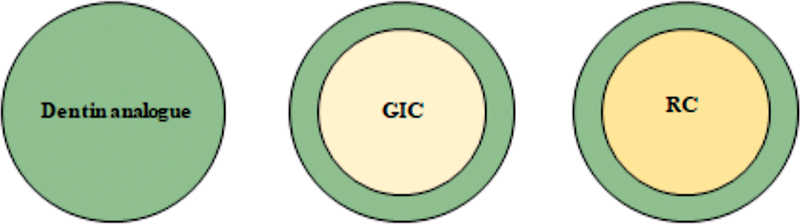
Occlusal view of the simulated foundation substrates. From the left to the right, the first condition simulated a substrate foundation only in dentin analogue. In the second condition, the substrate foundation has 1 mm axial walls in dentin analogue and the center in glass ionomer cement. The last condition presents 1 mm axial walls in dentin analogue and the center in resin composite.

This study simulated three different substrate surface conditions to bond the ceramic restorations: dentin analogue, dentin analogue + GIC, and dentin analogue + RC. The simulated base substrates are presented in Fig. 1 .

For the first foundation substrate simulation, a dentin analogue substrate (G10 epoxy resin, Protec, São Paulo, Brazil) was shaped in discs (2.6 mm × 10 mm diameter). Then, the discs were polished (#600 and #1200 grit silicon carbide papers) until the final thickness of 2.5 mm and cleaned with isopropyl alcohol in an ultrasonic bath (5 minutes). This setup was designed to reproduce an occlusal restoration for a posterior tooth. 10 A diameter of 10 mm was used to mimic the average area of a first molar. 19 For the other two groups, the preparation were kept as dentin substrate (axial walls with 1 mm thickness) while the center of the preparation was simulated containing different substrate materials: GIC or RC, similar to a class 1 restoration.

### Cementation

The intaglio surfaces of ceramic discs were etched with 5% hydrofluoric acid (Condac Porcelana 5%, FGM, Joinville, Brazil) for 20 seconds, washed by air-water spray, cleaned in an ultrasonic bath with distilled water for 5 minutes. The nthe silane agent (Monobond N, Ivoclar Vivadent) was applied for 20 seconds, left to react for 60 seconds, and gently air-dried. For the substrate bonding surface, the cementation surface was etched with 10% hydrofluoric acid (Condac Porcelana, FGM) for 60 seconds, followed by ultrasonic cleaning in distilled water (5 minutes) and the application of Multilink N Primer A and B mixture (Ivoclar Vivadent), using a microbrush under constant movement for 15 seconds and gentle air-drying for excess removal. Using the automix, the dual-cure resin cement (Multilink N, Ivoclar Vivadent) was applied on the center of the treated ceramic surfaces. 17

The discs (ceramic and substrate) were bonded with a standard load of 7.5 N on the occlusal surface of the ceramic, promoting uniform cement spreading. The excess cement was removed using a microbrush and light curing (high intensity of 1,000 mW/cm ^2^ ; wavelength ranging from 395 to 480 nm – Valo, Ultradent Products) was performed for 20 seconds on the occlusal surface of the ceramic, followed by 10 seconds in four points of the bonded interface. After 48 hours of immersion in distilled water, the specimens were submitted to aging simulation. 17

### Mechanical Cycling

**Fig. 2:**
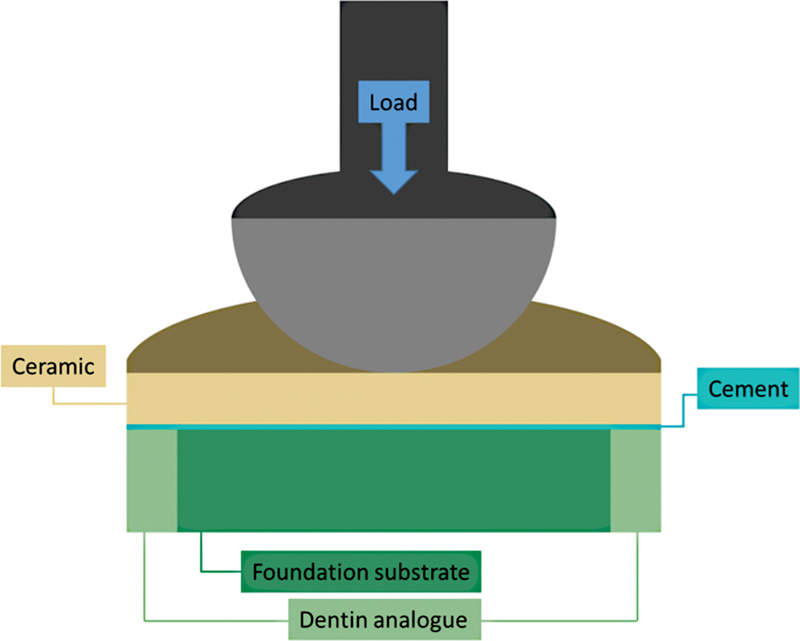
Schematic illustration of the cemented specimens submitted to the mechanical cycling and compression tests configuration.

All specimens were cyclic loaded vertically on the occlusal surfaces with 100 N. The fatigue test was set for 1 × 10 ^6^ cycles, at 4 Hz under water at 37°C. The loading was applied using a round stainless steel piston with 6 mm diameter. The specimens were inspected in every 100,000 cycles through the transillumination technique. Trough visual inspection, a unique light source with a fiber optic transilluminator was applied at 45 degrees to the ceramic surface. The light source was rotated until the perpendicular plane of the possible crack. If there was a crack, the light passage would be blocked and it would be visible. Failure was defined as large chippings, cracks, or ceramic bulk fracture. The specimen under mechanical cycling schematic illustration is presented in Fig. 2 .

### Thermocycling Aging

The specimens were submitted to mechanical and thermal cycling (5–37–55°C during 10,000 cycles) to investigate the behavior after long-term mission. Ten thousand thermocycles were performed since their correspondence to 1 year of *in vivo* environment. 20 Based on this information and for an optimized clinical situation, the thermal sequence included the human reference temperature of 37 °C between the cold and hot bath.

### Post-Fatigue Fracture Resistance

All specimens were tested positioned in a stainless steel base with their flat surface aligned perpendicular to the antagonist indenter in a universal testing machine (DL1000, EMIC) for the compressive test. The load was applied to the ceramic disc external surface using a unidirectional vertical device (1,000 kgf load cell, water) with a crosshead speed of 0.1 mm/min until failure occurred ( Fig. 2 ). The fracture load to start the failure (N) was computer recorded. Failed specimens were inspected under scanning electron microscopy (SEM) to identify the failure origin.

### Stress Calculation

For this study, the maximum center tensile stress was calculated using the FEA according to the foundation material and restoration thickness. Nevertheless, for three-layer specimens, the FEA results provide a good estimation of the maximum tensile stress at the center of the ceramic discs. 13 17 For this approach, the three-dimensional (3D) model of the *in vitro* assay was designed with the same dimensions as the testing specimens containing the ceramic disc, the cement layer, and foundation substrate. 13 17 The specimens' dimensions were converted from polylines to the 3D geometries modeling using a computer-aided software (Rhinoceros, version 5.0 SR8, McNeel North America). The final geometries were imported to the analysis software (ANSYS 17.2, ANSYS Inc.) in Standard for the Exchange of Product data format. Tetrahedral elements formed the mesh after a convergence test (10%) and each material's mechanical properties were assigned as having isotropic behavior. The elastic modulus and Poisson ratio used for the calculation were based on previous reports from the literature ( Table 2 ). 2 13 21 22 The compressive load of 100 N was used to perform an individual simulation and to obtain a specific value in MPa. The stress distribution data were exported and plotted as colorimetric stress maps. The tress peak was computed at the center of ceramic disc intaglio surface using the autoprobe from the software.

**Table 2:** Elastic modulus (E) and Poisson ratio ( *ν* ) of the materials used in the finite element analysis

Materials	E (GPa)	*v*
Composite resin 2	22.0	0.54
Glass Ionomer cement 11	8.0	0.25
Resin cement 18	9.2	0.30
Dentin analogue 18	18.0	0.30
Lithium disilicate 19	95.0	0.25

### Statistical Analysis

After the data normality verification using the Kolmogorov–Smirnov test, the *in vitro* data were analyzed by descriptive statistics and one-way analysis of variance (ANOVA) followed by multiple comparison post hoc Tukey test ( *α* = 0.05).

## Results

### Post-Fatigue Fracture Resistance

The mean fracture load and standard deviation per group are summarized in Table 1 . One-way ANOVA revealed statistical influence for the different groups ( *F* = 40.33; *p* < 0.001) on the average fracture load.

When the base substrate was the dentin analogue the pooled compressive resistance was 1,591 N, which was significantly higher than the pooled base substrates of GIC with 477 N or the RC base, with 1,115 N. The pooled differences between the GIC and the RC base were also significantly different. The thinner ceramic thickness showed statistical lower resistance (0.5 mm; 955 N) compared to the restoration in 1 mm, with 1,167 N.

**Fig. 3:**
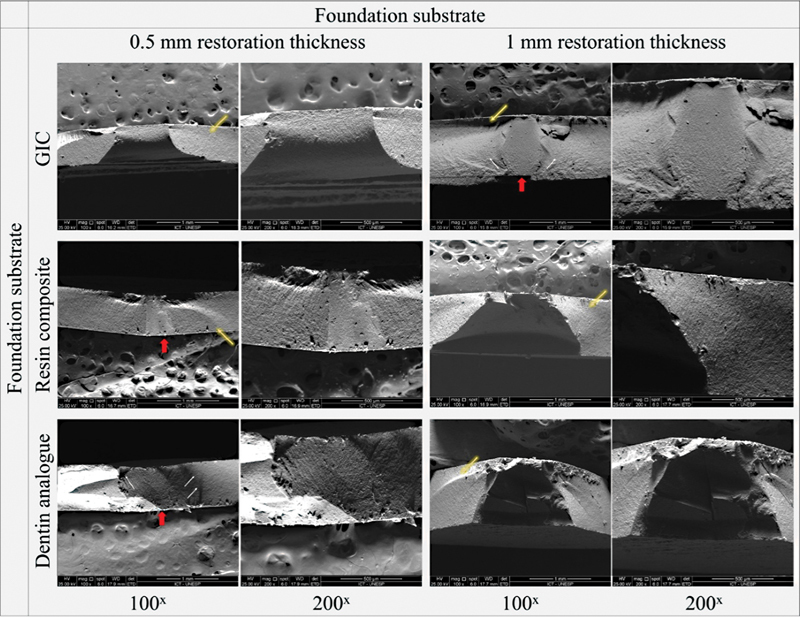
Scanning electron microscopy of representative failed specimens after the compressive test, under different magnifications. Red arrow presents the failure origin, white arrows indicate the direction of crack propagation, and yellow arrows, the compression curl. Failure originated from tensile side, however, as expected for a compressive load, damage caused by the load indenter was also observed, per example in the RC.5 specimen.

SEM revealed that the failure was originated in the tensile side ( Fig. 3 ). However, considerable damage was caused by the load indenter which was observed in many specimens.

### FEA Stress Calculation

**Fig. 4:**
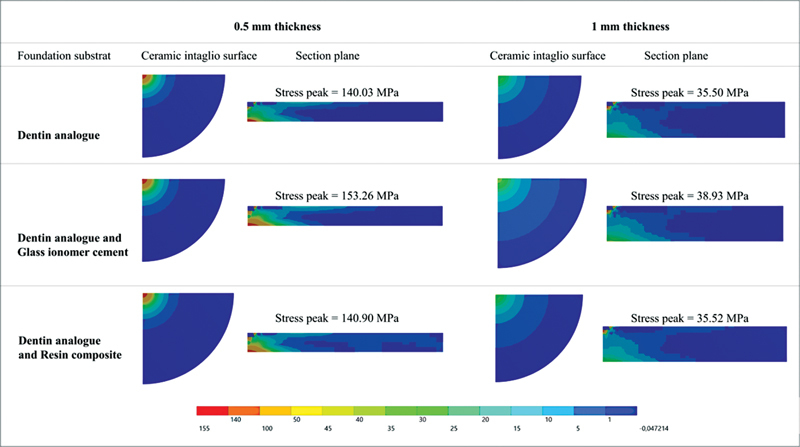
Maximum principal stress distribution map according to different substrates for 0.5 and 1.0 mm restoration thicknesses.

FEA revealed that the maximum principal stress ranged from 153.3 to 140.0 MPa in 0.5 mm restoration thickness. While for the thicker restoration (1.0 mm), the stress ranged from 35.5 to 38.9 MPa. Fig. 4 presents the colorimetric graph results. For both 0.5 and 1.0 mm restoration thickness, it is noticeable that the cementation in sound dentin analogue substrate promoted a reduced tensile stress magnitude. In addition, for foundation substrates in dentin with direct restorative material, the lower is the restorative material elastic modulus and the higher is the stress value. In this study, GIC promoted the highest stress peak in both 0.5 and 1.0 mm lithium disilicate restorations.

## Discussion

The present study revealed that there was an influence on the biomechanical behavior of adhesively cemented lithium disilicate restorations, according to the foundation substrate and ceramic thickness. The mean post-fatigue fracture resistance was higher in restorations cemented in the dentin analogues when compared to restored substrates (GIC or RC). In addition, the different ceramic thickness showed different behaviors when cemented onto the same foundation substrate material. Therefore, both hypotheses were accepted.

The ceramic fracture strength depends on the area of stress concentration during load application and its capacity to resist to the crack propagation from the region where the stress concentration takes place. 13 22 In addition, according to the results, the success of ceramic bonded to substrate depends basically on the restorative material stiffness, the type of substrate, and the bond strength between restoration and substrate. In the present study, fatigued lithium disilicate restorations presented an improved mechanical response and higher fracture-load when cemented onto dentin-like material in comparison to GIC. It was an expected behavior since the substrates with high stiffness reduce the flexural moment and consequently increases the load to fracture of adhesively cemented restorations. 10 11 13 This result is supported by the stress magnitude as demonstrated by FEA, where an increase in the first principal stress is observed to be inversely proportional to the foundation substrate elastic modulus.

The ceramic cemented on dentin concentrates less tensile stress on its surface, regardless of its thickness, which can be explained by the fact that this is the stiffer substrate simulated in this investigation. This result is in agreement with previous studies that evaluated different foundation materials suggesting that the lower is the stress at the adhesive interface, lower is the occurrence of catastrophic fractures in the restoration. 10 11 13

Despite the optimal performance from specimens cemented onto dentin substrate, the option to replace missing dentin tissue by dentin is still not possible. In vital teeth, small defects may be restored using GICs, whereas RC build-ups should be preferred for larger defects. 23 The use of GIC to support lithium disilicate ceramic material has been previously reported. 24 25 Some dentists prefer GICs for core build-ups due to its apparent ease of placement, fluoride release, good adherence, coefficient of thermal expansion similar to dentin, 26 and reduced residual stress. 14 In addition, GIC is a water-based material, so while still needing moisture control, it is not as technique-sensitive as RC. 26 On the other hand, composites have excellent strength and can be used to make core build-ups both for vital and nonvital teeth. Using RC, an effective bond between core and tooth are expected, however, only when moisture contamination and polymerization shrinkage can be properly controlled. 26 27

In short, when GIC was present, the lowest fracture load and the highest stress peaks were observed. However, according to the FEA the stress increases only 0.62% when GIC was used instead of RC for 0.5 mm restoration thickness, and 0.06% for 1.0 mm restoration thickness. Also, the average fracture load decreased in approximately 59% when GIC was used instead of RC for 0.5 mm restoration thickness, and 55% for 1.0 mm restoration thickness. Therefore, it seems plausible to assume that the inferior performance of GIC is mostly associated to the reduced bond strength instead only on the capability of load dissipation. Considering indirect restorations in which there is an interface between GIC and resin cement, the adhesion could be not so strong compared to an interface with chemically similar materials such as RC and resin cement. Supporting this hypothesis, it is possible to observe the bond strength data from previous studies, for example, the adhesion between a resinous material and GIC range between 3.8 until 4.4 MPa 27 ; while the bond strength between RC and resin cement can range from 13.7 until 31.2 MPa, 28 depending on the materials and pretreatment used. The reported bond strength values belong to different materials, tested in different conditions and environments, and were used only to illustrate the previous explanation.

In a clinical trial, the authors evaluated 36 fixed dental prosthesis made from a lithium disilicate glass ceramic placed in 28 patients. The restorations were cemented either with GIC or CR. The authors did not calculate the difference between the cementation protocols, but when GIC was used two recementations were needed during the 8-year follow-up while there was no debonding report for the adhesively luted group. 29 Therefore, the present study is in agreement with that when showing a lower resistance when GIC is present, justifying the high mechanical dampening effect at the restorative material caused by the low bond strength of GIC with resin cement.

The restoration's intaglio surface presents a significant effect at the ceramic longevity, in which a rough surface reduces the load to failure of indirect restorations. However, when a proper bonding was present this effect is inexistent. In addition, previous authors calculated that the tensile stress is homogeneously distributed in a bonded surface. 30 The present study corroborate with this statement, showing that the proper bonded conditions (dentin or RC) are better substrates than GIC. Considering 1.0 mm ceramics thickness bonded in dentin analogue, the previous study 30 calculated 1559.1 N fracture load for rough restorations while polished restorations showed higher resistance (1632.3 N). The values calculated in the present study were 1789 N for polished restorations bonded in dentin analogue while the presence of GIC as base material reduced the fracture load to 541 N. Apparently, not only the ceramic condition can affect the restoration longevity, but also the substrate stiffness and strong bondability.

Lithium disilicate restorations bonded in different foundation materials have been investigated previously. 31 32 The highest value of fracture resistance and survival rate for restorations were observed for partly bonded restorations to composite filling in comparison to dentin or enamel. 32 The present results showed less than 10% for stress peaks and similarity between fracture load for RC1.0 and D0.5. This shows that a RC is more promising than GIC when a filling material is necessary prior to the crown cementation. In addition, fracture features indicated the failures' origin in the adhesive interface 33 and the presence of cone cracks on the occlusal surface due to the compressive load application. With regards to the restoration thickness, higher stress magnitude was calculated in 0.5-mm groups, justifying that lower energy is necessary to fracture thin restorations, explaining the low fracture load values obtained in the *in vitro* test. On the other hand, there are previous investigations that concluded that the risk of fracture does not increase as the thickness of ceramic restorations decrease. 6 34 Similar to the present study, a previous *in vitro* investigation assessed the crown thickness effect on the fracture load of monolithic lithium disilicate. The loads to failure of the crowns were 369 N for 0.5 mm and 889 N for restorations with 1.0 mm. Therefore, the present result suggests that the minimally invasive dentistry concept with thinner restorations can be considered as a promising option when sound dentin or RC build-ups were present as substrate. Another study reported that the load-bearing capacity of specimen composed by ceramic/cement/dentin-like substrate tri-layer structure was approximately 872 N for static compressive loading for 1.0 mm of ceramic restoration. 35 36 This value is closer to the value found for RC base (1187 N), but lower than the average value for dentin substrate (1789 N). This difference could be explained due to the polishing protocol applied prior to the luting procedure.

A previous finite element study aimed to compare types of veneer preparations and their combination with three materials. 37 According to the authors, tooth structure, cement, and veneer showed equivalent values of total deformation comparing lithium disilicate glass ceramic and zirconia reinforced lithium silicate in all loading scenarios. This was justified by the similarity of elastic modulus between both materials. Despite that, the selection of a material with proper strength is mandatory, especially when considering thinner restorations as veneers with minimal preparation.

A clinical report described an 8-year follow-up evaluation using different thicknesses of porcelain laminate veneers in anterior teeth. 38 Nevertheless, after 6 years, this patient's adhesive interfaces were darkly stained, the patient was unsatisfied with the esthetics, and the porcelain was replaced by lithium disilicate glass ceramic. According to the authors, the use of lithium disilicate glass ceramic was preferable due to its association between esthetics and high strength. Additionally, the luting procedure when properly done can give significant support to the strength of the thin ceramic restorations. 40 Thermocycling is a commonly used fatigue method to evaluate bond durability, mimicking the thermal changes that occur in the oral cavity caused by eating, drinking, and breathing. 36 This method is valid to accelerate the restoration aging and to allow the long-term performance interpretation. Since the best aging protocol is still unclear, it is common to observe combined thermal and mechanical cycling to explain how degradation occurs and to give more details about the performance of adhesive systems. 36 Therefore, it is highly recommended that the restorative materials employed for restoring teeth should be capable enough to withstand such intraoral changes. 39 40 41 42

It is important to consider the present study's limitations despite the difference of the results calculated by the *in vitro* and *in silico* analyses: the test setup differs from the oral environment with different chewing forces, presence of sliding movements, parafunctional habits, and different antagonist materials. 42 Furthermore, different procedures that can affect the surface characteristics of ceramic materials like milling, etching, and inadequate polishing may increase the defects population inducing premature failure of the ceramic. 1 2 4 17 33 42 Therefore, the standardized specimens processing does not consider such variability. Considering the reported limitations, further clinical studies are still needed to confirm the present results.

## Conclusion

In clinical situations where a build-up core needs to be made prior to a prosthetic restoration in lithium disilicate, the core restorative material should be in RC instead of GIC. The RC's higher elastic modulus reduces the stress magnitude and increases the bond strength to the resin cement. When a core is not necessary, the bonding onto dentine shows better bond strength and biomechanical behavior. In addition, the thinner the restoration, the lower is the fracture load and the higher is the stress concentration.

## References

[1] Liu C Eser A Albrecht T Strength characterization and lifetime prediction of dental ceramic materials Dent Mater 2021 37 01 94 105 33208262 10.1016/j.dental.2020.10.015

[2] Borges A LS Costa A KF Dal Piva A MO Pinto A BA Tribst J PM Effect of three different veneering techniques on the stress distribution and in vitro fatigue behavior of core-veneer all-ceramic fixed partial dentures J Dent Res Dent Clin Dent Prospect 2021 15 03 188 196 10.34172/joddd.2021.032 PMC8538140 34712410

[3] Ausiello P Franciosa P Martorelli M Watts D C Numerical fatigue 3D-FE modeling of indirect composite-restored posterior teeth Dent Mater 2011 27 05 423 430 21227484 10.1016/j.dental.2010.12.001

[4] Feitosa F A Tribst J PM Araújo R M Pucci C R Surface etching and silane heating using Er: YAG and Nd: YAG lasers in dental ceramic luted to human dentin Int J Appl Ceram Technol 2021 18 1408 1416

[5] Matuda A GN Silveira M PM Andrade G S computer aided design modelling and finite element analysis of premolar proximal cavities restored with resin composites Materials (Basel) 2021 14 09 2366 34062936 10.3390/ma14092366 PMC8125402

[6] Srimaneepong V Heboyan A Zafar M S Fixed prosthetic restorations and periodontal health: a narrative review J Funct Biomater 2022 13 01 15 35225978 10.3390/jfb13010015 PMC8883934

[7] Gonzaga C C Cesar P F Miranda W G Jr Yoshimura H N Slow crack growth and reliability of dental ceramics Dent Mater 2011 27 04 394 406 21185074 10.1016/j.dental.2010.10.025

[8] Harada K Raigrodski A J Chung K-H Flinn B D Dogan S Mancl L A A comparative evaluation of the translucency of zirconias and lithium disilicate for monolithic restorations J Prosthet Dent 2016 116 02 257 263 26994676 10.1016/j.prosdent.2015.11.019

[9] Dal Piva A MO Tribst J PM Benalcázar Jalkh E B Anami L C Bonfante E A Bottino M A Minimal tooth preparation for posterior monolithic ceramic crowns: effect on the mechanical behavior, reliability and translucency Dent Mater 2021 37 03 e140 e150 33246664 10.1016/j.dental.2020.11.001

[10] Machry R V Borges A LS Pereira G KR Kleverlaan C J Venturini A B Valandro L F Influence of the foundation substrate on the fatigue behavior of bonded glass, zirconia polycrystals, and polymer infiltrated ceramic simplified CAD-CAM restorations J Mech Behav Biomed Mater 2021 117 104391 33618242 10.1016/j.jmbbm.2021.104391

[11] De Andrade G S Tribst J PM Orozco E I Influence of different post-endodontic restorations on the fatigue survival and biomechanical behavior of central incisors Am J Dent 2020 33 05 227 234 33017523

[12] Campaner L M Ribeiro A O Tribst J PM Loading stress distribution in posterior teeth restored by different core materials under fixed zirconia partial denture: a 3D-FEA study Am J Dent 2021 34 03 157 162 34143586

[13] Soares P M Cadore-Rodrigues A C Souto Borges A L Valandro L F Pereira G KR Rippe M P Load-bearing capacity under fatigue and FEA analysis of simplified ceramic restorations supported by Peek or zirconia polycrystals as foundation substrate for implant purposes J Mech Behav Biomed Mater 2021 123 104760 34418777 10.1016/j.jmbbm.2021.104760

[14] Ausiello P Dal Piva A MO Borges A LS Effect of shrinking and no shrinking dentine and enamel replacing materials in posterior restoration: a 3D-FEA Study Appl Sci (Basel) 2021 11 2215

[15] Sari T Ural C Yüzbasioglu E Duran I Cengiz S Kavut I Color match of a feldspathic ceramic CAD-CAM material for ultrathin laminate veneers as a function of substrate shade, restoration color, and thickness J Prosthet Dent 2018 119 03 455 460 28552290 10.1016/j.prosdent.2017.02.022

[16] Albelasy E Hamama H H Tsoi J KH Mahmoud S H Influence of material type, thickness and storage on fracture resistance of CAD/CAM occlusal veneers J Mech Behav Biomed Mater 2021 119 104485 33812289 10.1016/j.jmbbm.2021.104485

[17] Tribst J PM Dal Piva A MO Lopes G C Biaxial flexural strength and Weilbull characteristics of adhesively luted hybrid and reinforced CAD/CAM materials to dentin: effect of self-etching ceramic primer versus hydrofluoric acid etching J Adhes Sci Technol 2020 34 1253 1268

[18] de Jager N Münker T JAG Guilardi L F Jansen V J Sportel Y GE Kleverlaan C J The relation between impact strength and flexural strength of dental materials J Mech Behav Biomed Mater 2021 122 104658 34214922 10.1016/j.jmbbm.2021.104658

[19] Ferrario V F Sforza C Tartaglia G M Colombo A Serrao G Size and shape of the human first permanent molar: a Fourier analysis of the occlusal and equatorial outlines Am J Phys Anthropol 1999 108 03 281 294 10096680 10.1002/(SICI)1096-8644(199903)108:3<281::AID-AJPA4>3.0.CO;2-#

[20] Gale M S Darvell B W Thermal cycling procedures for laboratory testing of dental restorations J Dent 1999 27 02 89 99 10071465 10.1016/s0300-5712(98)00037-2

[21] Borges A LS Dal Piva A de O Moecke S E de Morais R C Tribst J PM Polymerization shrinkage, hygroscopic expansion, elastic modulus and degree of conversion of different composites for dental application J Compos Sci 2021 5 322

[22] Ma L Guess P C Zhang Y Load-bearing properties of minimal-invasive monolithic lithium disilicate and zirconia occlusal onlays: finite element and theoretical analyses Dent Mater 2013 29 07 742 751 23683531 10.1016/j.dental.2013.04.004 PMC3698988

[23] Naumann M Kiessling S Seemann R Treatment concepts for restoration of endodontically treated teeth: a nationwide survey of dentists in Germany J Prosthet Dent 2006 96 05 332 338 17098496 10.1016/j.prosdent.2006.08.028

[24] Ritzberger C Apel E Höland W Properties and clinical application of three types of dental glass-ceramics and ceramics for CAD-CAM technologies Materials (Basel) 2010 3 3700 3713

[25] Carvalho A O Bruzi G Anderson R E Maia H P Giannini M Magne P Influence of adhesive core buildup designs on the resistance of endodontically treated molars restored with lithium disilicate CAD/CAM crowns Oper Dent 2016 41 01 76 82 26266647 10.2341/14-277-L

[26] Field C Stone S Whitworth J Core build-ups and post placement. BDJ Clinician's Guides Springer Cham 2019 295 327

[27] Gopikrishna V Abarajithan M Krithikadatta J Kandaswamy D Shear bond strength evaluation of resin composite bonded to GIC using three different adhesives Oper Dent 2009 34 04 467 471 19678453 10.2341/08-009-L

[28] Dos Santos V H Griza S de Moraes R R Faria-E-Silva A L Bond strength of self-adhesive resin cements to composite submitted to different surface pretreatments Restor Dent Endod 2014 39 01 12 16 24516824 10.5395/rde.2014.39.1.12 PMC3916500

[29] Wolfart S Eschbach S Scherrer S Kern M Clinical outcome of three-unit lithium-disilicate glass-ceramic fixed dental prostheses: up to 8 years results Dent Mater 2009 25 09 e63 e71 19523678 10.1016/j.dental.2009.05.003

[30] de Kok P Pereira G KR Fraga S de Jager N Venturini A B Kleverlaan C J The effect of internal roughness and bonding on the fracture resistance and structural reliability of lithium disilicate ceramic Dent Mater 2017 33 12 1416 1425 29032826 10.1016/j.dental.2017.09.018

[31] Magne P Stanley K Schlichting L H Modeling of ultrathin occlusal veneers Dent Mater 2012 28 07 777 782 22575740 10.1016/j.dental.2012.04.002

[32] Sasse M Krummel A Klosa K Kern M Influence of restoration thickness and dental bonding surface on the fracture resistance of full-coverage occlusal veneers made from lithium disilicate ceramic Dent Mater 2015 31 08 907 915 26051232 10.1016/j.dental.2015.04.017

[33] Kelly J R Clinically relevant approach to failure testing of all-ceramic restorations J Prosthet Dent 1999 81 06 652 661 10347352 10.1016/s0022-3913(99)70103-4

[34] Holberg C Rudzki-Janson I Wichelhaus A Winterhalder P Ceramic inlays: is the inlay thickness an important factor influencing the fracture risk? J Dent 2013 41 07 628 635 23639702 10.1016/j.jdent.2013.04.010

[35] Yan J Kaizer M R Zhang Y Load-bearing capacity of lithium disilicate and ultra-translucent zirconias J Mech Behav Biomed Mater 2018 88 170 175 30173069 10.1016/j.jmbbm.2018.08.023 PMC6179910

[36] Amaral F LB Colucci V Palma-Dibb R G Corona S A Assessment of in vitro methods used to promote adhesive interface degradation: a critical review J Esthet Restor Dent 2007 19 06 340 353, discussion 354 18005284 10.1111/j.1708-8240.2007.00134.x

[37] Yousief S A Galal R M Alsharief H MA Comparison of two types of preparation for laminate veneer with three types of allceramic materials Eur J Dent 2023 17 01 120 126 35820443 10.1055/s-0042-1743143 PMC9949923

[38] Sá T CM de Carvalho M FF de Sá J CM Magalhães C S Moreira A N Yamauti M Esthetic rehabilitation of anterior teeth with different thicknesses of porcelain laminate veneers: an 8-year follow-up clinical evaluation Eur J Dent 2018 12 04 590 593 30369808 10.4103/ejd.ejd_241_17 PMC6178674

[39] Al-Ali A MAH Khalifa N Hadj-Hamou A Sheela S El-Damanhoury H M Effect of thickness and bonding technique on fatigue and fracture resistance of feldspathic ultra-thin laminate veneers Eur J Dent 2023 17 02 431 438 35728607 10.1055/s-0042-1745770 PMC10329525

[40] Ausiello P Ciaramella S Fabianelli A Mechanical behavior of bulk direct composite versus block composite and lithium disilicate indirect Class II restorations by CAD-FEM modeling Dent Mater 2017 33 06 690 701 28413061 10.1016/j.dental.2017.03.014

[41] Carvalho A O Bruzi G Giannini M Magne P Fatigue resistance of CAD/CAM complete crowns with a simplified cementation process J Prosthet Dent 2014 111 04 310 317 24388720 10.1016/j.prosdent.2013.09.020

[42] Durairaj R B Sivasaravanan S Sharma D K Ramachandran S Heboyan A Investigations on mechanical properties of titanium reinforced glass ionomer cement (GiC)—Ceramic composites suitable for dental implant applications Dig J Nanomater Biostruct 2021 16 161 167

